# And at the end, the Germans always win, don’t they? An evaluation of country-specific scoring behaviour in the dying seconds of international club soccer games

**DOI:** 10.1371/journal.pone.0202852

**Published:** 2019-04-16

**Authors:** Louis Van Den Broucke, Stijn Baert

**Affiliations:** 1 Ghent University, Ghent, Belgium; 2 Research Foundation–Flanders, Brussels, Belgium; 3 University of Antwerp, Antwerp, Belgium; 4 Universite´ Catholique de Louvain, Louvain-la-neuve, Belgium; 5 IZA, Bonn, Germany; 6 GLO, Maastricht, Netherlands; 7 IMISCOE, Rotterdam, Netherlands; Groupe ESC Dijon Bourgogne, FRANCE

## Abstract

This article contributes to the literature on performance determinants in soccer by investigating country differences in goal scoring in the dying seconds of international soccer games (i.e. in the 90th minute or later). We analyse this goal-scoring behaviour in 1,008 recent soccer games played in the Union of European Football Associations (UEFA) Champions League and Europa League. In contrast to Gary Lineker’s well-known quote that “at the end, the Germans always win”, no significant evidence is found for German teams scoring a goal in the dying seconds more often than other teams. Our results indicate, however, that European clubs do have an interest in learning from the end-of-game tactics used by French and Spanish clubs in recent international games as these teams were less likely to concede a goal during the dying seconds. English teams were also in this situation but only if they had an English coach.

## 1. Introduction

After losing the semi-final of the 1990 Fédération Internationale de Football Association (FIFA) World Cup against Germany, England’s former striker Gary Lineker declared the famous words “Soccer is a simple game: twenty-two men chase a ball for 90 minutes and at the end, the Germans always win” (source: https://www.brainyquote.com/quotes/quotes/g/garylineke422219.ht ml). This quote has become one of the most famous ones in international soccer. To date, it is widely used in online and print media. But is this statement true? The results of the FIFA World Cup through the years [1] show that Germany is not always the winner of this tournament. In a strict sense, then, we could reject the statement out of hand and end the article here. In a broader sense, however, Lineker’s quote can be interpreted as a hypothesis stating that German teams score a goal at the end of a soccer game (and thereby win the game) substantially more often than teams from other countries. In that respect, it is clear that different nations have their own traditions with respect to playing style [2], which indeed may have resulted in different scoring dynamics at the end of international soccer matches. If so, national and club teams may be willing to learn from the most successful traditions in this respect. Therefore, in the present study, we scientifically evaluate this country-specific, “nick-of-time” goal scoring and, thereby, evaluate the broad sense interpretation of Lineker’s theory.

We are not the first to investigate culture-related performance determinants in soccer. For instance, recently, Bachan, Reilly, and Witt [3] and Berlinschi, Schockkaert, and Swinnen [4] examined the impact of racial composition and players’ international mobility on the performance of national teams. However, our study is, by far, the closest related to that of van Ours and van Tuijl [2]. These authors directly investigated whether there are country-specific dynamics in goal scoring in the “dying seconds” of qualifying games for and/or tournament games at the European Championship, the Copa América, and the World Cup between 1960 and 2010—the dying seconds being defined by them as the 90th minute and added time of a soccer game. The main finding of their research was that, of the eight investigated national teams, Argentina, Germany, and Italy were more likely to score in the dying seconds of the analysed games. However, somewhat in contrast to Lineker’s theory, the German national team was also more likely to concede a goal at the end of these games. Therefore, van Ours and van Tuijl [2] interpret the country differences in goal scoring at the end of football games as country differences in risk seeking.

In the present study, we complement the research by van Ours and van Tuijl [2] for nation competitions by investigating country-specific goal scoring at the end of games in the world’s most important international tournaments for clubs, i.e. the Union of European Football Associations (UEFA) Champions League and UEFA Europa league, keeping clubs’ strength and other game characteristics constant. More concretely, we answer the following research questions.

- **Research question 1a (R1a).** Does the probability of scoring a goal in the dying seconds of an international soccer game differ by a club’s home country?
- **Research question 1b (R1b).** Does the probability of conceding a goal in the dying seconds of an international soccer game differ by a club’s home country?
- **Research question 1c (R1c).** Does the probability of winning a game based on goal scoring in the dying seconds of an international soccer game differ by a club’s home country?

On the one hand, based on the findings of van Ours and van Tuijl [2]—and based on Lineker’s prediction—a positive answer to R1a, R1b, and R1c can be expected. On the other hand, Kuper and Szymanski [5] argued (for the Turkish Süper Lig) that soccer culture is no longer affecting performance on the level of clubs, providing support for zero effects—or at least effects that are of a smaller magnitude than those found by van Ours and van Tuijl [2]. An important argument for this expectation given by Kuper and Szymanski [5] is that many club teams have a majority of players and/or a coach born in countries other than the team’s home country. To directly test this, in secondary analyses, we estimate whether country-specific scoring dynamics are moderated by whether the team coach and the majority of the players are of the same nationality as their club’s home country.

- **Research question 2a (R2a).** Is country-specific goal scoring in the dying seconds of a soccer game moderated by whether the majority of the team players are of the same nationality as the club’s home country?
- **Research question 2b (R2b).** Is country-specific goal scoring in the dying seconds of a soccer game moderated by whether the team coach is of the same nationality as the club’s home country?

We answer our research questions by analysing recent games played by clubs from the most prevalent countries in the UEFA Champions League and the UEFA Europa League. Besides Germany, these countries are England, Spain, Italy, France, Portugal, Russia, Ukraine, the Netherlands, and Belgium. More concretely, we analyse data for 1,008 soccer games played between 2008 and 2014.

By means of this research, we not only deepen the recent research on culture-related performance determinants in soccer but also, by extension, contribute to the scientific literature investigating success determinants in soccer generally [6–12].

The remainder of the article is organised as follows. In the second section, we describe our data and the methods that we used to analyse these data. In the third section, we present our research findings. In a final section, we draw our conclusions and offer suggestions for future research.

## 2. Methods

### 2.1. Data

To answer R1a, R1b, R1c, R2a, and R2b, we analysed all 1,008 soccer games in the group phase of the UEFA Champions League between 2008 and 2014 and the UEFA Europa League between 2011 and 2014—before 2011, another competition format was used for the UEFA Europa League. We analysed only games that were played in the group phase and not the games in the knock-out phase, because the notion of dying seconds is different for the latter phase. This is due to the fact that each round in the knock-out phase comprises a first and a second leg. In the first leg, the dying seconds are less crucial, as goals are summed up over the first and second leg. In the second leg, 30 minutes of extra time can be added when neither opponent scored more goals than the other one. These modalities may result in other game dynamics in the 90th minute and (regular) added time of these first and second legs compared to games in the group phase. We return to this point at the end of Section 3. For more information on the general set-up of the analysed competitions, we refer the reader to the UEFA’s official website (http://www.uefa.com).

Following Ponzo and Scoppa [13] and van Ours and van Tuijl [2], we used each game twice in our dataset, once from the perspective of the home team and once from the perspective of the away team, resulting in 2,016 observations at the team-game level. To take into account the related outcomes for both observations at the level of the game, in our analyses we clustered the standard errors at the game level. In addition, as a robustness check, we randomly assigned each game either to the home or the visiting team (and thereby considered each game only once). However, this did not change our research conclusions.

More concretely, our dataset results from merging the game data constructed by Baert and Amez [6] with data from other sources. The data of Baert and Amez [6] comprise a large set of game characteristics and events for all aforementioned games in the UEFA Champions League and the UEFA Europa League based on the UEFA’s online reports. Table 1 provides the average value of the variables that were used in our regression analyses. Panel A presents the variables used as dependent variables in one or more analyses, panel B shows the main independent variables, and panel C includes the other game characteristics that are (mainly) used as control variables.

**Table 1:** Data: Summary statistics.

	Mean
***A*. *Dependent variables***	
Goal scored in dying seconds	0.086
Goal conceded in dying seconds	0.086
Winning the game	0.375
***B*. *Independent variables***	
Team is English	0.101
Team is Spanish	0.092
Team is Italian	0.080
Team is German	0.077
Team is French	0.071
Team is Portuguese	0.060
Team is Russian	0.054
Team is Ukrainian	0.048
Team is Dutch	0.042
Team is Belgian	0.036
Team from Central Europe	0.173
Team from Northern Europe	0.045
Team from Eastern Europe	0.110
Team from South-east Europe	0.170
Team from Southern Europe	0.232
Team from Western Europe	0.271
***C*. *Control variables***	
Majority of players from team are of same nationality as team	0.444
Coach is of same nationality as team	0.614
Two goals or more ahead after 89th minute	0.185
One goal ahead after 89th minute	0.188
Score is equal after 89th minute	0.253
One goal behind after 89th minute	0.189
Two goals or more behind after 89th minute	0.185
Home team	0.500
Relative strength	0.000
Game in UEFA Europa League	0.423
Game with no importance for team	0.131
Game with no importance for opponent	0.131
*N*	2,016

Notes: A definition of these variables can be found in Section 2.

In line with van Ours and van Tuijl [2], we used an indicator of whether the team in question scored a goal in the dying seconds of the game as our benchmark dependent variable. An alternative label for these dying seconds is “Cesarini Time”, in reference to the former Italian international soccer player Renato Cesarini’s habit of scoring late [14]. As previously mentioned, we define scoring a goal in the dying seconds as scoring a goal in the 90th minute or later—soccer games usually last a little bit longer than 90 minutes because the referee can add time, correlated with the time the gameplay had been stopped due to injuries or substitutions. So, our benchmark dependent variable is 1 in case the team in question scores during these dying seconds, and 0 otherwise. As a consequence of this definition, if both teams score a goal in the dying seconds—which was the case in only 3 out of the 1,008 sampled games—this indicator is 1 for both observations related to this game (i.e. the observations for the home and away team). The average value of our benchmark dependent variable is 0.086, meaning there is an 8.6% probability for each team to score in the dying seconds of a game. In other words, there was a goal in the dying seconds in 174 (i.e. 17.2% = 2 × 8.6%) of the analysed games. For answering R1a, we will investigate whether this variable differs between different countries, keeping other game characteristics and events (up to the 89th minute) constant.

To answer R1b and R1c, two additional variables were constructed. First, we made up an indicator of whether the team in question conceded a goal in the dying seconds of the game, using a definition analogous to the one for our benchmark dependent variable. The average value for this first alternative dependent variable is also 0.086, which is logical given the fact that each game is used twice in our data: a goal scored by a team results in a goal conceded by his opponent. Second, we included an indicator of whether the team in question won the game. The latter alternative dependent variable is, on average, 0.375, which implies that there was a draw in 25.0% (i.e. 1–2 × 0.375) of the analysed games.

We answer our research questions with respect to the 10 countries with the highest number of games played in our data (and, thereby, in the UEFA Champions League and UEFA Europa League during the considered time window). As shown in panel B of Table 1, in 10.1% of our team-game observations the team is English, i.e. the country that is the most often represented. In 3.6% of the observations, the team is Belgian, i.e. the country that is the 10th most often represented. The total share of the 10 most prevalent countries is 66.1%, so that in 33.9% of the observations the team is from a country other than those 10.

To be able to answer R2a and R2b, we enriched the data of Baert and Amez [6] with two variables derived from the information on worldfootball.net (http://www.worldfootball.net), i.e. (i) a continuous variable capturing how many of the team’s 11 players who started the game were of the same nationality as that of the team, and (ii) an indicator of whether the nationality of the team coach corresponded to the nationality of the team. As can be seen from panel C of Table 1, in only 44.4% of the team-game level observations, the majority of the starting players (i.e. six or more) were of the same nationality as the team, while in 61.4% of the observations the nationality of the coach corresponded to that of the team. By interacting the mentioned country indicators with (i) and (ii), we obtained restricted versions of our benchmark independent variables, i.e. a team is seen as being from a particular country only if enough players or the coach were born in that country.

Besides these (restricted) country dummies, we also constructed a set of region indicators as alternative independent variables. More concretely, these dummy variables enabled us to also test whether goal-scoring behaviour during the dying seconds is, potentially, country-specific as well as region-specific. The lower rows of panel B show that, in our data, the team is from Central Europe (Austria, Czech Republic, Germany, Hungary, Poland, Slovakia, Slovenia, or Switzerland) in 17.3% of the observations; from Northern Europe (Denmark, Norway, or Sweden) in 4.5% of the observations; from Eastern Europe (Belarus, Russia, or Ukraine) in 11.0% of the observations; from South-east Europe (Azerbaijan, Bulgaria, Croatia, Cyprus, Greece, Israel, Kazakhstan, Macedonia, Romania, Serbia, or Turkey) in 17.0% of the observations; from Southern Europe (Italy, Spain, or Portugal) in 23.2% of the observations; and from Western Europe (Belgium, England, France, Ireland, the Netherlands, or Scotland) in 27.1% of the observations.

Finally, we included some other game characteristics and events that may correlate both with the mentioned dependent and independent variables and, therefore, serve in our analyses as controls. First, we condition on the score at the end of the 89^th^ minute of the game, as captured by indicators of five situations in which the team might be, just before the dying seconds: (i) two goals or more ahead, (ii) one goal ahead, (iii) score is equal, (iv) one goal behind, and (v) two goals or more behind at the end of the 89th minute. Second, we control for more regular determinants of success in soccer games: “home” status, relative strength of the team and of its opponent, an indicator of games in the UEFA Europa League, and indicators of games with no importance for the team and its opponent [6, 15, 16]. The “relative strength” variable is, in line with Baert and Amez [6], defined as the natural logarithm of the quotient of the team and its opponent’s UEFA coefficient for the season in question plus 1 (to avoid division by 0 for teams who did not participate in the UEFA Champions League or UEFA Europa League during the five previous seasons, as the UEFA coefficient of a team is based on its participation and results in these seasons). In addition, in line with Baert and Amez [6], games with no importance for a team are defined as games in which it is mathematically impossible for this team to change its qualification status for the next round. This is the case if a team is sure it will finish the group stage: (i) as winner or runner-up of its group in the UEFA Champions League or UEFA Europa League; (ii) in third place in its group in the UEFA Champions League; (iii) in fourth place in its group in the UEFA Champions League; or (iv) in either third place or fourth place in its group in the UEFA Europa League.

The data are available as S1 File.

### 2.2. Econometric approach

These data were analysed by means of linear probability models. As previously mentioned, we clustered the standard errors at the game level. As a consequence, our regression analyses are also robust to heteroscedasticity, which is important given the binary nature of our dependent variables [17]. In addition, we looked into the corresponding results when replacing these models with logistic models. The estimated marginal effects for the latter models were very similar to the results presented in the next section.

## 3. Results

Given Lineker’s quote concerning the German soccer team(s), in a first analysis, we focus on the scoring behaviour of the German teams in our data only. In other words, we answer R1a, R1b, and R1c when simplifying country differences to differences between German and non-German teams. The results of this analysis can be found in Table 2. In regression model (1), we regress our benchmark dependent variable, i.e. an indicator of goal scoring by the team during the dying seconds, on an indicator for German teams only. From model (2) on, we condition on the score at the end of the 89th minute, and from model (3) on, we add the other control variables mentioned in the previous section. Models (4) and (5) are the same as model (3), but with the indicators of “a goal conceded in the dying seconds” and of “winning the game” as alternative dependent variables, respectively. Following Lineker’s quote, a significantly positive effect of the indicator of German teams is expected for all of these models except for model (4), where a significantly negative effect is expected. However, in none of the models was a significant effect found.

**Table 2:** Results: Benchmark model.

	(1)	(2)	(3)	(4)	(5)
**Team is German**	**0.018 (0.025)**	**0.012 (0.025)**	**0.013 (0.025)**	**0.002 (0.023)**	**0.003 (0.016)**
Two goals or more ahead after 89th minute		0.024 (0.019)	0.020 (0.019)	-0.015 (0.016)	0.927*** (0.011)
One goal ahead after 89th minute		0.049** (0.020)	0.047** (0.021)	0.018 (0.019)	0.844*** (0.018)
Score is equal after 89th minute (reference)					
One goal behind after 89th minute		0.012 (0.018)	0.018 (0.019)	0.047** (0.021)	-0.068*** (0.011)
Two goals or more behind after 89th minute		-0.023 (0.016)	-0.015 (0.017)	0.020 (0.019)	-0.068*** (0.011)
Home team			0.003 (0.013)	-0.003 (0.013)	0.005 (0.009)
Relative strength			0.008** (0.003)	-0.008** (0.003)	0.003 (0.003)
Game in UEFA Europa League			-0.000 (0.012)	-0.001 (0.012)	-0.002 (0.008)
Game with no importance for team			-0.021 (0.020)	-0.028 (0.020)	-0.017* (0.010)
Game with no importance for opponent			-0.028 (0.020)	-0.021 (0.020)	0.008 (0.011)
Intercept	0.085*** (0.006)	0.074*** (0.012)	0.077*** (0.015)	0.081*** (0.015)	0.070*** (0.013)
Dependent variable: Goal scored in dying seconds	Yes	Yes	Yes	No	No
Dependent variable: Goal conceded in dying seconds	No	No	No	Yes	No
Dependent variable: Winning the game	No	No	No	No	Yes
*N*	2,016	2,016	2,016	2,016	2,016

Notes: The presented statistics are linear (probability) regression model estimates. The estimation results for the model’s independent variables are in bold. A definition of the variables adopted in the regressions can be found in Section 2. Standard errors, which are adjusted for 1,008 clusters on the level of the game, are between parentheses.

***, **, and * indicate significance at the 1%, 5%, and 10% significance level, respectively.

In a second analysis, we re-estimate our most extensive model (in terms of control variables included) for a larger set of country indicators as our independent variables and, thereby, answer R1a, R1b, and R1c from a broader perspective. More concretely, in models (1)–(3) of Table 3, we predict our three dependent variables by means of a set of country dummies for the four most prevalent countries (i.e. England, Spain, Italy, and Germany) and in models (4)–(6), we do the same using the full set of 10 country dummies. We find for none of these countries a significantly higher chance of scoring a goal in the dying seconds.

**Table 3:** Results: Multiple team nationality indicators as main explanatory variables.

	(1)	(2)	(3)	(4)	(5)	(6)
**Team is German**	**0.018 (0.025)**	**-0.004 (0.024)**	**0.002 (0.016)**	**0.028 (0.026)**	**-0.012 (0.025)**	**-0.003 (0.016)**
**Team is English**	**0.017 (0.024)**	**-0.000 (0.022)**	**-0.013 (0.017)**	**0.027 (0.025)**	**-0.009 (0.023)**	**-0.018 (0.018)**
**Team is Spanish**	**0.023 (0.025)**	-0.031* (0.019)	**0.008 (0.016)**	**0.038 (0.027)**	-0.040* (0.021)	**0.003 (0.017)**
**Team is Italian**	**-0.003 (0.024)**	**-0.017 (0.022)**	**-0.003 (0.016)**	**0.008 (0.025)**	**-0.025 (0.024)**	**-0.009 (0.016)**
**Team is French**				**0.042 (0.030)**	-0.050** (0.022)	**0.010 (0.018)**
**Team is Portuguese**				**0.010 (0.027)**	**-0.022 (0.027)**	**-0.005 (0.017)**
**Team is Russian**				**0.030 (0.031)**	**0.002 (0.032)**	**-0.015 (0.021)**
**Team is Ukrainian**				**-0.004 (0.029)**	**-0.000 (0.032)**	**-0.001 (0.021)**
**Team is Dutch**				**0.009 (0.032)**	**-0.007 (0.033)**	-0.061*** (0.023)
**Team is Belgian**				**0.012 (0.035)**	**0.018 (0.039)**	**0.010 (0.020)**
**Team is from other country (reference)**						
Two goals or more ahead after 89th minute	0.016 (0.019)	-0.013 (0.017)	0.927*** (0.012)	0.016 (0.020)	-0.012 (0.017)	0.927*** (0.012)
One goal ahead after 89th minute	0.046** (0.021)	0.018 (0.019)	0.844*** (0.018)	0.045** (0.021)	0.020 (0.019)	0.844*** (0.018)
Score is equal after 89th minute (reference)						
One goal behind after 89th minute	0.020 (0.019)	0.045** (0.021)	-0.068*** (0.011)	0.020 (0.019)	0.046** (0.021)	-0.070*** (0.011)
Two goals or more behind after 89th minute	-0.012 (0.017)	0.018 (0.020)	-0.068*** (0.011)	-0.012 (0.017)	0.018 (0.020)	-0.069*** (0.011)
Home team	0.004 (0.013)	-0.004 (0.013)	0.005 (0.009)	0.004 (0.013)	-0.004 (0.013)	0.005 (0.009)
Relative strength	0.008** (0.003)	-0.008** (0.003)	0.003 (0.003)	0.007* (0.004)	-0.007** (0.004)	0.004 (0.003)
Game in UEFA Europa League	0.002 (0.013)	-0.004 (0.013)	-0.002 (0.008)	0.006 (0.013)	-0.008 (0.013)	-0.002 (0.009)
Game with no importance for team	-0.023 (0.020)	-0.025 (0.020)	-0.018** (0.010)	-0.024 (0.021)	-0.023 (0.020)	-0.019* (0.010)
Game with no importance for opponent	-0.026 (0.020)	-0.023 (0.020)	0.009 (0.011)	-0.025 (0.020)	-0.025 (0.020)	0.010 (0.010)
Intercept	0.071*** (0.016)	0.088*** (0.016)	0.071*** (0.013)	0.060*** (0.018)	0.096*** (0.019)	0.076*** (0.014)
Dependent Variable: Goal scored in dying seconds	Yes	No	No	Yes	No	No
Dependent Variable: Goal conceded in dying seconds	No	Yes	No	No	Yes	No
Dependent Variable: Winning the game	No	No	Yes	No	No	Yes
*N*	2,016	2,016	2,016	2,016	2,016	2,016

Notes: The presented statistics are linear (probability) regression model estimates. The estimation results for the model’s independent variables are in bold. A definition of the variables adopted in the regressions can be found in Section 2. Standard errors, which are adjusted for 1,008 clusters on the level of the game, are between parentheses.

***, **, and * indicate significance at the 1%, 5%, and 10% significance level, respectively.

However, with regard to conceding a goal in these dying seconds, we do find some country-specific patterns. Both in models (2) and (5), a weakly significantly smaller chance to concede a goal in the dying seconds is found for Spanish teams. According to model (5), they are 4.0 percentage points less likely to concede such a late goal (*p* = 0.056). In addition, French teams are found to have a 5.0 percentage point lower chance of conceding a goal in the dying seconds (*p* = 0.027).

Finally, with respect to winning the game conditional on the score at the end of the 89th minute, a highly significantly negative effect is found for Dutch teams. More concretely, they are 6.1 percentage points less likely to win the game, keeping the situation at the start of the dying seconds constant (*p* = 0.007). This might be surprising, as the Dutch teams do not have a substantially higher chance of conceding (or scoring) a goal in the dying seconds. Further analysis shows, however, that both observations are compatible: Dutch teams often go from both one goal ahead or one goal behind at the end of the 89th minute to a tie at full time.

Table 4 presents the results of a third analysis in which we replace the country dummies of the former analysis with the region dummies mentioned in Section 2. In other words, we answer R1a, R1b, and R1c by broadening our view from country differences to regional differences in goal scoring. However, we do not find a significant effect of any of the region indicators on any of the dependent variables. So, the region of the country where the team is located has no effect on the team’s goal-scoring behaviour in the dying seconds or its ability to eventually win the game based on these late goals.

**Table 4:** Results: Team region indicators as main explanatory variables.

	(1)	(2)	(3)
**Team from Northern Europe**	**-0.038 (0.026)**	**0.026 (0.038)**	**0.007 (0.014)**
**Team from Eastern Europe**	**-0.009 (0.024)**	**0.018 (0.025)**	**-0.014 (0.016)**
**Team from South-east Europe**	**-0.023 (0.020)**	**0.011 (0.022)**	**-0.005 (0.011)**
**Team from Southern Europe**	**-0.004 (0.021)**	**-0.017 (0.019)**	**-0.004 (0.013)**
**Team from Western Europe**	**-0.002 (0.021)**	**-0.000 (0.019)**	**0.013 (0.013)**
**Team from other region (reference)**			
Two goals or more ahead after 89th minute	0.018 (0.019)	-0.013 (0.016)	0.927*** (0.011)
One goal ahead after 89th minute	0.046** (0.021)	0.019 (0.019)	0.843*** (0.018)
Score is equal after 89th minute (reference)			
One goal behind after 89th minute	0.019 (0.019)	0.045** (0.020)	-0.069*** (0.011)
Two goals or more behind after 89th minute	-0.014 (0.017)	0.018 (0.020)	-0.069*** (0.011)
Home team	0.003 (0.013)	-0.003 (0.013)	0.005 (0.008)
Relative strength	0.007** (0.004)	-0.007** (0.004)	0.004 (0.003)
Game in UEFA Europa League	0.002 (0.012)	-0.005 (0.012)	-0.003 (0.009)
Game with no importance for team	-0.021 (0.020)	-0.025 (0.020)	-0.018* (0.010)
Game with no importance for opponent	-0.027 (0.020)	-0.023 (0.020)	0.008 (0.010)
Intercept	0.085*** (0.021)	0.082*** (0.019)	0.078*** (0.016)
Dependent Variable: Goal scored in dying seconds	Yes	No	No
Dependent Variable: Goal conceded in dying seconds	No	Yes	No
Dependent Variable: Winning the game	No	No	Yes
*N*	2,016	2,016	2,016

Notes: The presented statistics are linear (probability) regression model estimates. The estimation results for the model’s independent variables are in bold. A definition of the variables adopted in the regressions can be found in Section 2. Standard errors, which are adjusted for 1,008 clusters on the level of the game, are between parentheses.

***, **, and * indicate significance at the 1%, 5%, and 10% significance level, respectively.

Finally, we answer R2a and R2b by replicating models (1)–(3) in Table 3 when using the restricted versions of the four country indicators. More concretely, in models (1)–(3) of Table 5, we regress the three dependent variables on restricted indicators of English, Spanish, Italian, or German teams, which take, as mentioned in Section 2, only value 1 in case not only the team is based in England, Spain, Italy, or Germany, but also a majority of this team’s players is of English, Spanish, Italian, or German nationality, respectively. In these regression models, we also add a control variable capturing the general effect of playing with a majority of players of the same nationality (as the team). Models (4)–(6) are quite similar to these three models, except for the fact that the country dummies take value 1 exclusively if the coach is also of the same nationality as the team. The only significant country-related effect found in these analyses is that an English team led by an English coach is 7.1 percentage points less likely to concede a goal in the dying seconds of the analysed games (*p* = 0.000). So, the lower probability of conceding a goal in the dying seconds for Spanish teams becomes insignificant when using the restricted country dummies.

**Table 5:** Results: Interactions with nationality of players and coach as main explanatory variables.

	(1)	(2)	(3)	(4)	(5)	(6)
**Team is German × Majority of team players are German**	**0.036 (0.036)**	**-0.016 (0.030)**	**0.026 (0.021)**			
**Team is English × Majority of team players are English**	**-0.030 (0.055)**	**-0.036 (0.056)**	**-0.003 (0.014)**			
**Team is Spanish × Majority of team players are Spanish**	**0.041 (0.037)**	**-0.033 (0.025)**	**0.026 (0.023)**			
**Team is Italian × Majority of team players are Italian**	**0.028 (0.047)**	**-0.022 (0.035)**	**0.008 (0.033)**			
**Team is German × Coach is German**				**0.005 (0.027)**	**0.016 (0.029)**	**0.005 (0.017)**
**Team is English × Coach is English**				**0.001 (0.059)**	-0.071*** (0.012)	**-0.002 (0.011)**
**Team is Spanish × Coach is Spanish**				**0.036 (0.033)**	**-0.022 (0.024)**	**0.016 (0.022)**
**Team is Italian × Coach is Italian**				**0.014 (0.028)**	**-0.011 (0.024)**	**0.011 (0.018)**
Two goals or more ahead after 89th minute	0.018 (0.019)	-0.014 (0.017)	0.926*** (0.011)	0.020 (0.019)	-0.014 (0.017)	0.927*** (0.011)
One goal ahead after 89th minute	0.046** (0.020)	0.018 (0.019)	0.843*** (0.018)	0.047** (0.021)	0.017 (0.019)	0.844*** (0.018)
Score is equal after 89th minute (reference)						
One goal behind after 89th minute	0.019 (0.019)	0.046** (0.021)	-0.068*** (0.011)	0.019 (0.019)	0.046** (0.021)	-0.067*** (0.011)
Two goals or more behind after 89th minute	-0.013 (0.017)	0.019 (0.020)	-0.067*** (0.011)	-0.014 (0.017)	0.020 (0.020)	-0.066*** (0.011)
Home team	0.004 (0.013)	-0.003 (0.013)	0.005 (0.008)	0.003 (0.013)	-0.003 (0.013)	0.005 (0.009)
Relative strength	0.008** (0.003)	-0.008** (0.003)	0.003 (0.003)	0.008** (0.003)	-0.008** (0.003)	0.003 (0.002)
Game in UEFA Europa League	0.003 (0.013)	-0.001 (0.013)	-0.001 (0.008)	0.001 (0.013)	0.000 (0.013)	-0.000 (0.008)
Game with no importance for team	-0.023 (0.020)	-0.026 (0.020)	-0.018* (0.010)	-0.022 (0.020)	-0.027 (0.020)	-0.017* (0.010)
Game with no importance for opponent	-0.027 (0.020)	-0.022 (0.020)	0.009 (0.011)	-0.027 (0.020)	-0.020 (0.020)	0.008 (0.011)
Majority of players from team are of same nationality as team	-0.008 (0.014)	-0.002 (0.015)	0.002 (0.009)			
Coach is of same nationality as team				0.000 (0.014)	-0.007 (0.015)	-0.005 (0.009)
Intercept	0.076*** (0.015)	0.085*** (0.015)	0.066*** (0.013)	0.074*** (0.017)	0.087*** (0.016)	0.070*** (0.014)
Dependent Variable: Goal scored in dying seconds	Yes	No	No	Yes	No	No
Dependent Variable: Goal conceded in dying seconds	No	Yes	No	No	Yes	No
Dependent Variable: Winning the game	No	No	Yes	No	No	Yes
*N*	2,016	2,016	2,016	2,016	2,016	2,016

Notes: The presented statistics are linear (probability) regression model estimates. The estimation results for the model’s independent variables are in bold. A definition of the variables adopted in the regressions can be found in Section 2. Standard errors, which are adjusted for 1,008 clusters on the level of the game, are between parentheses.

***, **, and * indicate significance at the 1%, 5%, and 10% significance level, respectively.

To test the robustness of the presented analyses, we conducted some additional regressions of which the results are available on request. First, we replicated our analyses when using the probability of losing the game (instead of winning the game) as dependent variable. Second, we re-estimated all models after including expenditures on transfers between season 2008–2009 and season 2013–2014, derived from information on Transfermarkt.de (http://www.transfermarkt.de), as an addition control variable capturing the teams’ economic power. This control was not included in our main analyses as it may be vulnerable to reverse causality problems: game outcomes and transfer decisions are endogenous. Finally, we also analysed the 714 games in the knock-out phase. As mentioned in Section 2, these games were excluded from the data for our main analyses. However, these sensitivity analyses led to similar conclusions as those described in the previous paragraphs.

We end this section by mentioning some secondary research findings related to the control variables adopted throughout our regression models. Firstly, we find a significantly higher probability of scoring a goal in the dying seconds for teams who are one goal ahead after the 89th minute of the game. A possible explanation for this may be that, in such a case, the team’s opponent has to take high risks to avoid losing the game, potentially leading to more space and opportunities for the team that already has the lead. Consistent with this, we also find a significantly higher probability of conceding a goal in the dying seconds for teams who are trailing with one goal at the end. Secondly, we do not find evidence for a home advantage in scoring (and not conceding) goals in the dying seconds of international football games. Thirdly, in line with Bachan et al. [3], no evidence is found for (i) a team’s composition by nationality of the players or (ii) its coach’s nationality affecting the scoring behaviour in the dying seconds.

## 4. Conclusion

We contributed to the scientific literature investigating success determinants in soccer in general and the recent subliterature on culture-related performance determinants in soccer by investigating country differences in goal-scoring behaviour in the dying seconds of international club soccer games. More concretely, we investigated whether: (i) the probability of scoring (or conceding) a goal in the dying seconds of a soccer game differs by a club’s country and (ii) whether this country-specific goal scoring is moderated by whether the majority of the team players and the team coach are of the same nationality as the club’s country. We found that in the 1,008 analysed recent soccer games in the group phase of the UEFA Champions League and the UEFA Europa League, French and Spanish teams were less likely to concede a goal during the dying seconds. In addition, Dutch teams were more likely to end the game in a tie as a consequence of (scoring or conceding) a late goal. Finally, English teams were less likely to concede a late goal, but only if they had an English coach. In contrast to Gary Lineker’s quote with which we started this article, we did not find significant evidence for German teams scoring (or conceding) a goal more often in the dying seconds. Thus, our findings for these international club competitions differ from those reported by van Ours and van Tuijl [2] for nation competitions.

Our results indicate that European clubs from outside of France and Spain may have an interest in learning from the end-of-game tactics used by French and Spanish teams in the recent seasons of the European international club competitions. Whether these teams’ success in avoiding late goals during recent seasons is related to real country-level factors or to team-level factors could be the subject of follow-up research. In particular, we are in favour of future research that investigates particular country-level characteristics (such as wealth, demographics, and climate) as drivers of performance in soccer.

## Supporting information

S1 File (XLSX)
